# Case Report: Myelodysplastic syndrome- associated myeloid sarcoma: an unusual clinical presentation of a rare disease

**DOI:** 10.12688/f1000research.7899.1

**Published:** 2016-02-02

**Authors:** Emoke Horvath, Smaranda Demian, Elod Nagy

**Affiliations:** 1 Deptartment of Pathology, University of Medicine and Pharmacy of Targu Mures, Targu-Mures, Romania; 2 Hematology Clinic 1, Emergency Clinical Hospital,, University of Medicine and Pharmacy of Targu Mures, Targu-Mures, Romania; 3 Deptartment of Pharmacy and Biochemistry, University of Medicine and Pharmacy of Targu Mures, Targu-Mures, Romania; 4, Laboratory of Medical Analysis, Clinical County Hospital Mures, Targu-Mures, Romania

**Keywords:** myeloid sarcoma, myelodysplastic syndrome, myeloid blasts, immunophenotype

## Abstract

Myeloid sarcoma results from the extramedullary homing and proliferation of immature myeloid precursors. We present the timeline, events and diagnostic pitfalls related to a 66 year-old male patient’s case, admitted to the Hematology Clinic for pancytopenia, fever, weight loss and fatigue. The severe cytopenia and the few blasts observed in his blood smear indicated a bone marrow biopsy. The bone marrow showed hypercellularity and multilineage dysplasia with the presence of 15% myeloblasts. After the biopsy, he promptly developed paraplegia and nuclear magnetic resonance revealed an epidural tumour which was then resected.In the epidural tumour mass blast-like, round cells were observed with a complex immunophenotype, characterized by myeloperoxidase, CD117, CD15, CD99, leucocyte common antigen positivity and a high Ki-67 proliferation index. Considering the main differential diagnostic issues, the final diagnosis was stated as myelodysplastic syndrome-associated myeloid sarcoma. The prognosis was unfavourable, the bone marrow was quickly invaded by proliferating blast cells, and despite chemotherapy attempts, the patient died.

## Introduction

Myeloid sarcoma is a rare malignant solid tumour resulting from the extramedullary proliferation of myeloblasts or immature myeloid cells, that usually precedes or accompanies acute myeloid leukaemia (AML), chronic myeloproliferative disorders, sometimes presenting as the first sign of AML ^1^. Every anatomical site of the body can be involved, but most common are the soft tissues of the head and neck, bone, skin and less often the genitourinary tract, the central nervous system and the spinal cord ^2, 3^. We present a case with a complex clinical manifestation resulting from the unusual localization of the tumour mass, and that coincides with the MDS involving simultaneously the bone marrow tissue.

## Case presentation

An otherwise healthy, 66-year-old man was admitted to the Haematology Clinic of the Emergency County Hospital (Targu-Mures, Romania) in a poor general condition, presenting fatigue, continuous fever, night sweating and weight loss (10 kg in 3 months), with symptoms occurring for a few weeks. His past medical history was unremarkable, without a known history of toxic or drug exposure. Physical examination evidenced a mucocutaneous pallor, but no lymphadenopathy or organomegaly. Acute myeloid leukaemia/myelodysplastic syndrome was suspected. Baseline laboratory data confirmed symptoms of ineffective haematopoiesis. Peripheral blood examination revealed pancytopenia: severe anaemia, trombocytopenia and leukopenia with 2% blasts of myeloid lineage ( Table 1), associated with elevated serum LDH (1250 U/L, reference range 240–480 U/L). The low-level presence of blasts in the blood smear (2%) indicated a bone marrow biopsy in order to establish the diagnosis, which was effected on the second day of hospitalization. Post-biopsy, after a few hours he developed paraplegia. Cerebrospinal nuclear magnetic resonance was recommended and performed, which elucidated an epidural tumour mass of the thoracic spine (suspected metastasis), compressing the dural sac in the spinal channel at the T1–T3 levels. No other tumoral formation was found by complex imagistic investigation. On the same day, the patient underwent an emergent laminectomy, the tumour being totally resected. The bone marrow biopsy and the tumour fragments were submitted for histopathological examination.

**Table 1.** The complete blood count values of the patient.

CBC	Results	Reference range of the laboratory
Haemoglobin	74	120–160 g/L
MCV	104	80–95 fL
Platelet count	78	150–400 ×10 ^9^/L
WBC	2.1	3.5–10.0 ×10 ^9^/L
Neutrophils	19%	40–75%
Lymphocytes	74%	19–48%
Monocytes	5%	3–9%
Blasts	2%	0%

The hypercellular bone marrow biopsy showed a multilineage dysplasia characterized by dyserythropoiesis, dysgranulopoiesis and dysmegakaryopoiesis with approximately 15% CD34/CD117 positive myeloblasts without Auer rods, associated with the presence of abnormal localisation of immature precursors (ALIP). Based on this histologic picture, according to the clinical and laboratory data, our diagnosis was refractory anaemia with excess blasts-2 (RAEB-2). The spinal tumour fragments showed a diffuse proliferation of undifferentiated myeloblast-like round tumour cells, mixed with a few neutrophil precursors.

Considering the two histological findings, myeloid sarcoma was suspected, but for a differential diagnosis lymphoblastic lymphoma, Burkitt lymphoma, diffuse large B-cell lymphoma and small round cell tumours also had to be excluded. A large immunohistochemistry panel was helpful in achieving differentiation of these conditions. The monomorphic tumour cells revealed positive stainings for Leucocyte Common Antigen (LCA), myeloperoxidase (MPO), CD15 and CD117, associated with a high Ki67 index (over 60%). Neuroendocrine and lymphoid markers, pan CK and also CD68 were negative. In contrast, a significant number of tumour cells expressed CD99 antigen.

The histopathological diagnosis has been stated on the basis of the two biopsies, being completed to “ Myelodysplastic syndrome (RAEB-2) associated myeloid sarcoma” of the spinal cord.

The clinical course was unfavourable, at the end of the first week post-surgery, the peripheral blood and bone marrow suddenly showing a burst of the blast count up to 50%. Chemotherapy with high-dose cytosine arabinoside proved to be inefficient, and the patient died at 5 weeks from admission.

**Figure 1.**
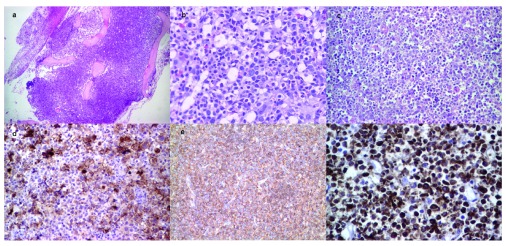
( **a**– **f**). Hypercellular bone marrow biopsy with reduced fatty cells, hematoxylin and eosin staining, magnification 4X ( **a**), in the central portion of the bone marrow with foci of ALIP and dysplastic features in granulocytic lineage, hematoxylin and eosin staining, magnification 20X ( **b**). Spinal tumor mass with diffuse and infiltrative population of large malignant cells (myeloblasts), with abundant cytoplasm and large nuclei, hematoxylin and eosin staining, magnification 10X ( **c**), many of them with CD99, magnification 20x ( **d**), and CD45 antigen expression, magnification 10X ( **e**). Tumour cells showing strong MPO positivity, magnification 20X, 2', 3-diaminobenzidin (DAB) immunostaining ( **f**).

## Discussion

The exact prognosis of myeloid sarcoma is difficult to determine, but in general is poor ^1^. The first description of “myeloid sarcoma” appeared in literature in 1811, and in 1904 its association with acute leukaemia was identified ^4^.

The term "granulocytic sarcoma” was introduced in 1967 by Rappaport to describe only tumours of granulocytic origin, however the term is now often applied to any tumour related to acute leukaemia or myelodysplastic syndrome (MDS) ^5^.

The mechanisms of extramedullary involvement are not fully understood, but it was demonstrated that leukaemic cell surface markers (CD56) are implicated in extramedullary homing ^5^.

The particularity of our case consists of the unusual tumour localization with spinal compression and neurologic signs, and the combination with the deficient haematopoiesis, that raises the suspicion of a metastasis in the bone marrow. Regarding phenotype, the positivity for CD45 and CD99 with a high Ki-67 proliferation index could suggest a lymphoma, but the morphological similarities between the extramedullary tumour cells and the bone marrow blasts are suggestive for myeloid sarcoma. For an accurate approach, a large-scale immunohistochemistry panel, containing mandatorily the CD45, MPO, CD15, CD79a, CD3, CD34, CD117, CD99, TdT and Ki-67 proved to be useful. The major diagnostic pitfalls are represented by distinction from lymphoblastic lymphomas (TdT+/CD34+/CD99+/CD45+/high Ki-67 index), and small round-cell tumours (CD99+/TdT-). Seifert *et al.* analyzed 12 cases of myeloid sarcoma and found a consistent positivity for CD117, CD43, MPO, CD68, CD34 and a sporadic reaction of leucocyte common antigen ^6^. Two major issues of the correct diagnostic orientation are the recognition of a possible non-lymphoid CD45 positivity and ruling-out of the aberrant CD99 antigen from the pivotal markers in this tumour type. On the other hand, MPO positivity is a prominent feature for a tumour of myeloid origin that overrides the unusual and aberrant antigen expression (CD79a, CD99).

In conclusion, we considered, that a large panel of antibodies must be used to establish the challenging diagnosis of myeloid sarcoma. This condition that should always be considered as a diagnostic possibility in patients with MDS, AML or chronic myeloproliferative disorders complicated with a secondary tumor mass.

## Consent

Written informed consent was obtained from the patient for the management and publication of clinical and laboratory data. The Ethical Committee of the Hospital approved the publication of the clinical details (no. 780/14-01-2016).
