# Supplementary material for: Case Report: Myelodysplastic syndrome- associated myeloid sarcoma: an unusual clinical presentation of a rare disease
Source: F1000Res. 2016 Feb 2;5:128. [Version 1] doi: 10.12688/f1000research.7899.1 (PMC4805157; doi:10.12688/f1000research.7899.1)
Supplement: Peer review history

# Peer review: Referee response for version 1

**DOI:** 10.5256/f1000research.8503.r12978

**Published:** 2016-03-22

**Authors:** Erika Toth

**Affiliations:** 1 Surgical and Molecular Tumour Pathology Centre, National Institute of Oncology, Budapest, Hungary

Well-structured paper. Rare case, nice demonstration of the difficult differential diagnostic process of myeloid sarcoma. The title is appropriate and represent the work. Results supports the final conclusion of the case.

I have read this submission. I believe that I have an appropriate level of expertise to confirm that it is of an acceptable scientific standard.

# Peer review: Referee response for version 1

**DOI:** 10.5256/f1000research.8503.r12783

**Published:** 2016-03-14

**Authors:** Judit Csomor

**Affiliations:** 1 1st Department of Pathology and Experimental Cancer Research , Faculty of Medicine, Semmelweis University, Budapest, Hungary

The main aim of this short case report is to create attention for myeloid sarcoma, which can be a difficult differential diagnostic problem, first of all in cases, when the myeloid sarcoma precedes the bone marrow disease. The documentation of the presented patient illustrates well the histological work up and the diagnosis, but the paper would have been more teaching if the authors add more detail about the patients course. I would have been interested in the results of investigation of the cerebrospinal fluid, the cytogenetic findings and the neurological status after operation? Why monotherapy was chosen? What further complications did they observe? What was the cause of death?

In my experience myeloid sarcoma can be purely blastic, but frequently shows signs of myeloid  differentiation. Fine cytoplasmic granulation of the immature cells, presence of eosinophilic myelocytes among the blasts, or some monocytoid features are suspicious for myeloid tumor, and can be recognised by careful examination of high quality sections and special stains. If these signs are observed a short panel containing the lineage specific markers is enough to confirm the diagnosis  For differential diagnosis of a poorly differentiated tumor, the authors list a wide panel of immune stains emphasizing markers of immaturity (CD34, CD117, tdt, CD99). I think the lineage specific markers, especially CD45, MPO, CD3, CD79, Pax5 and lysozyme, are more important. To suggest a cost effective algorithm in the report would be a valuable supplement.

And I have to add, that the diagnosis of MDS with excess of blasts and AML is first of all based on bone marrow smears and flow cytometry. If flow cytometry is not available cytochemistry is recommended for defining the cell lineage of the blasts. Bone biopsy is usually only an additional investigation (except of dry tap). Probably in the presented case smears also were elaborated, but data of smears and bone marrow biopsy is not clearly separated. The blast count is based on CD34 and CD117 immunohistochemistry without mentioning the ratio of the blast by marrow smear cytology.  The authors state, that CD56 may have role in homing of the myeloid blasts, therefore additional CD56 investigation would be interesting. AML with t(8;21) is more frequently cause myeloid sarcoma, therefore applying FISH probes to test the genotype of the tumor is also recommended to get more information about these rare tumors.

I have read this submission. I believe that I have an appropriate level of expertise to confirm that it is of an acceptable scientific standard.
